# Water Extracts of Hull-less Waxy Barley (*Hordeum vulgare* L.) Cultivar ‘Boseokchal’ Inhibit RANKL-induced Osteoclastogenesis

**DOI:** 10.3390/molecules24203735

**Published:** 2019-10-16

**Authors:** Kwang-Jin Kim, Yongjin Lee, So-Ri Son, Hyunjin Lee, Young-Jin Son, Mi-Kyung Lee, Mija Lee

**Affiliations:** 1 Department of Pharmacy, Sunchon National University, Suncheon 57922, Korea; mastiffk@naver.com (K.-J.K.); yojilee@gmail.com (Y.L.); 2 Department of Biomedical Science and technology, Graduate School, Kyung Hee University, Seoul 02447, Korea; allosori@khu.ac.kr; 3 Department of Crop Foundation, National Institute of Crop Science (NICS), Rural Development Administration (RDA), Wanju 55365, Korea; poi7346@korea.kr; 4 Department of Food and Nutrition, Sunchon National University, Jeonnam, Suncheon 57922, Korea

**Keywords:** osteoporosis, bone, osteoclast, barley, extracts, NFATc1

## Abstract

Osteoporosis is a disease that leads to reduced bone mineral density. The increase in patient and medical costs because of global aging is recognized as a problem. Decreased bone mass is a common symptom of bone diseases such as Paget’s disease, rheumatoid arthritis, and multiple myeloma. Osteoclasts, which directly affect bone mass, show a marked increase in differentiation and activation in the aforementioned diseases. Moreover, these multinucleated cells made from monocytes/macrophages under the influence of RANKL and M-CSF, are the only cells capable of resorbing bones. In this study, we found that the water extracts of Boseokchal (BSC-W) inhibited osteoclast differentiation in vitro and investigated its inhibitory mechanism. BSC-W was obtained by extracting flour of Boseokchal using hexane and water. To osteoclast differentiation, bone marrow-derived macrophage cells (BMMs) were cultured with the vehicle (0.1% DMSO) or BSC-W in the presence of M-CSF and RANKL for 4 days. Cytotoxicity was measured by CCK-8. Gene expression of cells was confirmed by real-time PCR. Protein expression of cells was observed by western blot assay. Bone resorption activity of osteoclast evaluated by bone pit formation assay using an Osteo Assay Plate. BSC-W inhibited RANKL-induced osteoclastogenesis in a dose-dependent manner without exerting a cytotoxic effect on BMMs. BSC-W decreased the transcriptional and translational expression of c-Fos and NFATc1, which are regulators of osteoclastogenesis and reduced the mRNA expression level of TRAP, DC-STAMP, and cathepsin K, which are osteoclast differentiation marker. Furthermore, BSC-W reduced the resorption activity of osteoclasts. Taken together, our results indicate that BSC-W is a useful candidate for health functional foods or therapeutic agents that can help treat bone diseases such as osteoporosis.

## 1. Introduction

Bones are dynamic tissues of various types of cells that undergo regeneration and repair processes known as bone remodeling. Osteoclasts and osteoblasts are cells that play an important role in bone maintenance [1], and the balance between osteoclastic and osteoblastic activity is important to maintenance of bone homeostasis. Increased numbers or activity of osteoclasts in bone homeostasis causes bone loss in diseases such as osteoporosis, Paget’s disease, rheumatoid arthritis, and periodontal disease [2].

Osteoporosis, which is the most common bone disease in the world, is associated with bone mass decrease and fracture risk. Patients with osteoporosis have a low bone density and weakened microstructure, which is likely to lead to pathological fractures. Fractures caused by osteoporosis are recognized as a global public health problem [3]. Such fractures can cause considerable pain and severe disability, leading to poor quality of life. The main cause of osteoporosis is related to increased osteoclast numbers and their bone resorption activity [3]. 

Osteoclasts are tartrate-resistant acid phosphatase-positive multinuclear cells (TRAP^+^-MNCs) formed by the fusion of several mononuclear precursors that are responsible for bone resorption. Osteoclasts remove old or weak bones, and are involved in maintenance of blood mineral levels [4,5]. Osteoclast differentiation is regulated by two cytokines, macrophage colony stimulating factor (M-CSF) and receptor activator of nuclear factor-κB (NF-κB) ligand (RANKL) [6]. M-CSF produced by osteoblasts (or immune cells) induces the expression of RANK and the survival signal of osteoclasts in osteoclast precursor cells [7]. RANKL secreted by osteoblast/activated T cells binds to the osteoclast receptor RANK and induces the activation of mitogen-activated protein kinase (MAPK), c-Fos, and NFATc1 [5,6,8,9,10]. NFATc1, the key transcription factor for osteoclast formation, regulates the expression of osteoclast differentiation and activation factors such as TRAP, cathepsin K, and dendritic cell-specific transmembrane protein (DC-STAMP) [11,12,13,14,15].

Studies of the development of fruit or grains containing functional ingredients and antioxidants have been conducted by many researchers all over the world. Anthocyanin is known to be an antioxidant with antidiabetic activity, as well as cholesterol lowering, anticancer, and anti-aging effects [16,17,18,19]. Recently, Boseokchal was developed as a new barley strain with a high content of anthocyanin [20,21]. In this study, we investigated the effects of Boseokchal extract on osteoclast differentiation and activation directly related to osteoporosis.

## 2. Results

### 2.1. BSC-W Inhibited RANKL-Mediated Osteoclast Differentiation

**Figure 1:**
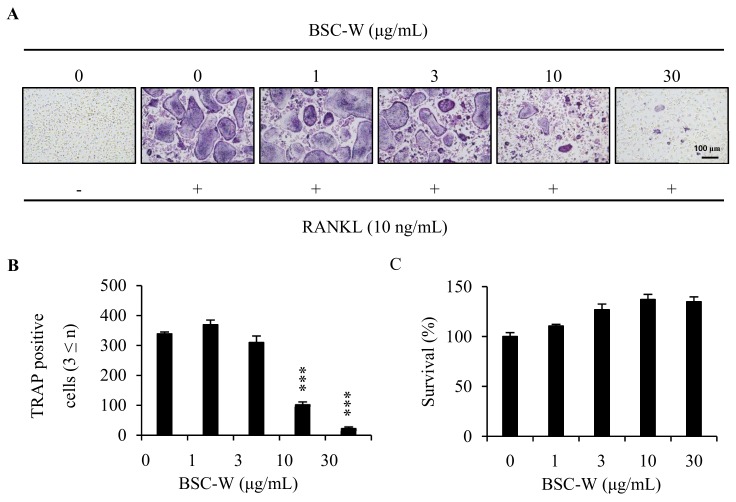
BSC-W inhibited osteoclast differentiation. (**A**) BMMs were cultured with 10 ng/mL RANKL and 30 ng/mL M-CSF for 4 days in the presence of vehicle (0.1% DMSO) or the indicated concentrations of BSC-W. Cells were fixed in 3.7% formalin, permeabilized with 0.1% Triton X-100, and stained with TRAP solution. (**B**) TRAP-positive multinucleated cells (3 or more nuclei) were counted as osteoclasts. *** p < 0.001 (*n* = 3). (**C**) BMMs were cultured with 30 ng/mL M-CSF for 3 days in the presence of vehicle (0.1% DMSO) or the indicated concentrations of BSC-W. The effects of BSC-W on BMMs viability were assessed using a CCK-8 assay kit (*n* = 3).

To examine whether BSC-W affected RANKL-mediated osteoclastogenesis, BMMs were cultured with RANKL and M-CSF in the presence of 0.1% DMSO (vehicle) or BSC-W (0, 1, 3, 10, 30 μg/mL) for 4 days. BMMs were differentiated into TRAP^+^-MNCs by RANKL, but BSC-W significantly reduced this differentiation (Figure 1A). When the number of TRAP^+^-MNCs with three or more nuclei was analyzed, the formation of osteoclasts was significantly decreased at a BSC-W concentration of more than 10 μg/mL (Figure 1B).

### 2.2. BSC-W Had No Cytotoxic Effect On Bmms

To determine whether inhibition of osteoclastogenesis was due to cytotoxicity by BSC-W, we conducted cytotoxicity studies in BMMs. Briefly, BMMs were cultured in 10% α-MEM treated with 0.1% DMSO (vehicle) or BSC-W (1, 3, 10, 30 μg/mL) for 3 days in the presence of 30 ng/mL M-CSF. As shown in Figure 1C, BSC-W did not exert cytotoxicity toward BMMs at the concentrations used in this study (Figure 1C).

### 2.3. Effects of BSC-W on RANKL-Induced Gene Expression 

**Figure 2:**
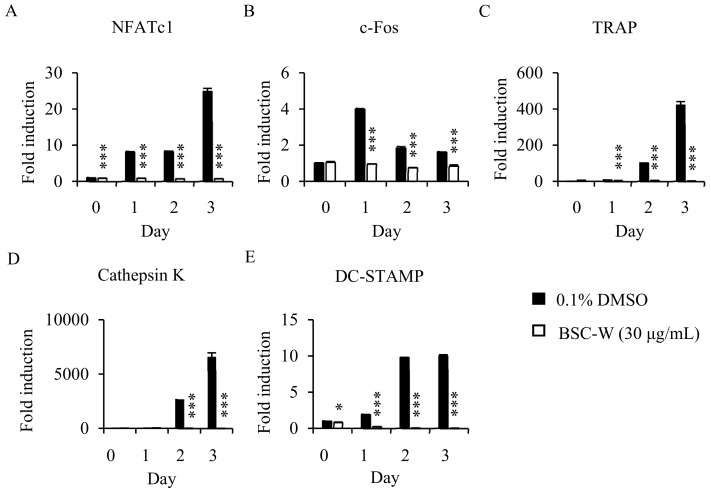
Effects of BSC-W on RANKL-mediated mRNA expression of NFATc1. BMMs were treated with vehicle (0.1% DMSO) or BSC-W (30 μg/mL) and 30 ng/mL M-CSF for 1 h and then 10 ng/mL RANKL at the indicated times. Total RNA was subsequently isolated using TRIzol reagent, after which the mRNA expression levels were evaluated by real-time PCR. (**A**) NFATc1, (**B**) c-Fos, (**C**) TRAP, (**D**) Cathepsin K, and (**E**) DC-STAMP were used. Glyceraldehyde-3-phosphate dehydrogenase (GAPDH) was used as the internal control. *, p < 0.05; ***, p < 0.001.

To confirm the mechanism of inhibition activity of BSC-W in osteoclast differentiation, we analyzed the expression of c-Fos and NFATc1, transcription factors that regulates osteoclastogenesis, and the marker genes involved in osteoclast differentiation, such as TRAP, DC-STAMP, and cathepsin K. RANKL increased the level of mRNA expression of c-Fos and NFATc1, but BSC-W significantly decreased their expression level (Figure 2A,B). BSC-W also significantly reduced the mRNA expression level of TRAP, DC-STAMP, and cathepsin K (Figure 2C–E).

### 2.4. BSC-W Inhibited RANKL-Induced Protein Expression of NFATc1

**Figure 3:**
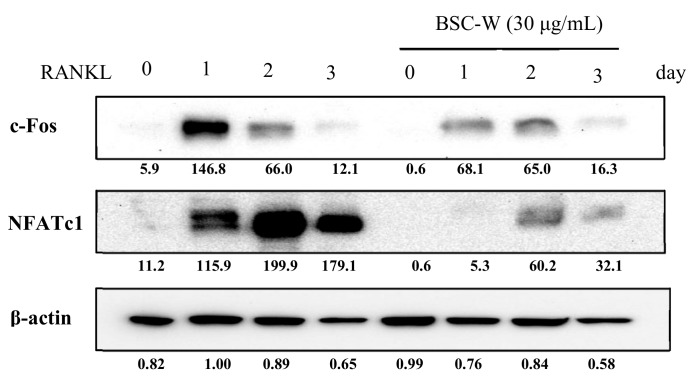
BSC-W decreases RANKL-mediated protein expression of NFATc1. BMMs were pretreated with vehicle (0.1% DMSO) or BSC-W (30 μg/mL) and 30 ng/mL M-CSF for 1 h prior to 10 ng/mL RANKL stimulation at the indicated times. Cell lysates were resolved by SDS-PAGE, and western blotting was performed with anti-c-Fos, anti-NFATc1, and anti-actin antibodies as indicated. The above figures of c-Fos and NFATc1 were calibrated based on the loading amounts of β-actin.

We confirmed the effects of BSC-W on protein expression of c-Fos and NFATc1 using Western blot analysis. The protein expression of c-Fos and NFATc1 induced by RANKL was significantly decreased by BSC-W (Figure 3). These results indicated that BSC-W inhibited NFATc1 expression and osteoclast formation.

### 2.5. Effects of BSC-W on RANKL-Mediated Bone Resorptive Activity of Osteoclasts

**Figure 4:**
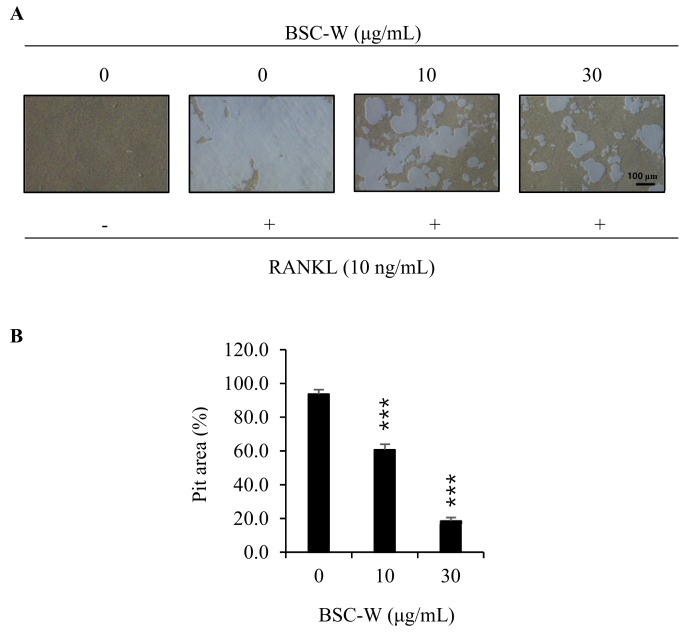
BSC-W inhibited bone resorption by RANKL-induced osteoclasts. (**A**) BMMs were plated on an Osteo Assay Plate and treated with 30 ng/mL M-CSF and 10 ng/mL RANKL in the presence of different concentrations of BSC-W. After 4 days of culture, the cells attached to the Osteo Assay Plate were removed and photographed under a light microscope. (**B**) Pit areas were quantified using the Image J program. *** *p* < 0.001 (*n* = 3).

We investigated whether BSC-W affects bone resorption of osteoclasts. The osteoclasts made a wide pit area on the bone slice, but BSC-W drastically reduced the pit area in a dose-dependent manner (Figure 4).

## 3. Discussion

Osteoporosis is a disease in which the quality of the bone deteriorates and fracture rate increases. In 2000, a total of 9 million osteoporotic fractures occurred, including 1.6 million pelvic fractures, 1.7 million forearm fractures, and 1.4 million vertebral fractures [22]. The increase in osteoporosis patients because of population aging has become a problem for the health and economy of the world [23,24]; therefore, investigation of osteoporosis is necessary for the prevention and treatment of osteoporosis.

Bone quality is greatly influenced by the balance between the activity of bone-resorbing osteoclasts and bone-forming osteoblasts. Osteoclasts, which originate from mononuclear cells/macrophages of bone marrow, are the only cells that can absorb bone [25]. These cells are produced and matured through various steps, such as differentiation and fusion, and the overall process is regulated by the RANKL-RANK signaling system [5]. Specifically, RANKL-RANK signaling is important to the formation of osteoclasts and for the treatment of pathological bone loss by these cells. After RANKL binds to the receptor, RANK, it rapidly activates MAPKs signaling molecules, such as p38, ERK, and JNK, and eventually promotes the activation of c-Fos-NFATc1 signaling, which is known to be an important transcription factor in osteoclast differentiation and activation [26,27,28].

Barley, which seems to have been cultivated in the Mesopotamia region since 7000 BC, is one of the major grains, along with rice, wheat, sorghum, and corn. Barley and wheat are believed to be the first crops grown by humans. In the previous studies, β-D-glucan, a representative dietary fiber of barley, decreased blood cholesterol levels in the body [29,30]. The value of barley as a healthy food is gradually increasing because of its excellent nutritional materials. The Boseokchal used in this study is an improved strain of hull-less waxy barley that contains high levels of the antioxidant anthocyanin and has therefore attracted attention as a functional food.

Here, we investigated the effects of Boseokchal’s water extract (BSC-W) on RANKL-induced osteoclastogenesis. BSC-W significantly reduced the differentiation of RANKL-treated macrophages into osteoclasts with no cytotoxicity, confirming that it inhibited osteoclast formation. Transcriptional factors of the NFAT family are known for being key molecules involved in the regulation of various biological systems, as well as in the formation of osteoclasts [31,32,33,34]. Therefore, we analyzed the expression levels of NFATc1 mRNA and protein by real-time PCR and western blotting, respectively, to check the influence of BSC-W against osteoclast differentiation. BSC-W significantly inhibited the expression levels of RANKL-induced NFATc1 mRNA and protein during osteoclast differentiation. In addition, mRNA expression of TRAP, DC-STAMP, and cathepsin K, which are differentiation/activation markers of osteoclasts, were significantly decreased by BSC-W. We also confirmed that the bone resorptive activity of osteoclasts was inhibited by BSC-W using a pit assay.

In previous papers, polyphenols and flavonoids, which are the major components of plant extracts, they are known as the inhibitive materials against osteoclast differentiation in osteoporosis. So it would be helpful in the treatment of osteoporosis [35,36,37,38,39,40]. According to the results of analyzing the contents of polyphenol and flavonoid component in BSC-W, the total polyphenol content was 8.73 ± 0.05 mg gallic acid equivalents/g and the total flavonoid content was 41.27 ± 1.46 mg quercetin equivalents/g. Therefore, it was confirmed that the inhibitive activity of BSC-W against the differentiation and resorption of osteoclasts caused by polyphenols and flavonoids. 

Taken together, these results suggested that BSC-W could prevent osteoclast differentiation by inhibiting the expression of NFATc1 and the activities of osteoclastogenic-related factors during osteoclast formation and differentiation. Therefore, BSC-W could be applied as a useful functional food or therapeutic agent for bone diseases such as osteoporosis.

## 4. Materials and Methods

### 4.1. Preparation of the Boseokchal Water Extract

Preparation of Boseokchal water extract was performed as previously described [41]. Briefly, flour of Boseokchal was defatted three times with 1 L hexane for 24 h at room temperature. After filtration with Whatman No. 3, the residue was extracted three times with 1 L prethanol, then passed through a Büchner funnel lined with filter paper (Carl Roth, Karlsruhe, Germen, 111A, Ø100 mm). The residue of the prethanol extraction was subsequently extracted twice with 1 L of water for 24 h at room temperature, after which the liquid was concentrated on a rotary evaporator and lyophilized to give an extract. The total flavonoid and phenolic contents of BSC-W was analyzed by the method of previous papers [42,43].

### 4.2. Cell Culture and Osteoclast Differentiation

In vitro cell experiments were performed as previously published paper [44]. All cells were cultured at 37 °C under 5% CO_2_ and medium was renewed every three days. Bone marrow cells (BMCs) were obtained from the femurs and tibiae of five week-old male ICR mice (n = 2: Raon Bio, Yongin, Korea). The protocol was approved by the Sunchon National University Institutional Animal Care and Use Committee (SCNUIACUC; Permit No. SCNU IACUC 2016-06). The BMCs from mice were cultured with 10 ng/mL macrophage colony-stimulating factor (M-CSF; Peprotech, Rocky Hill, NJ, USA) overnight in α-MEM (Thermo Fisher Scientific Inc., Waltham, MA, USA) containing 10% fetal bovine serum (FBS; Thermo Fisher Scientific Inc., Waltham, MA, USA) and 100 U/mL penicillin/streptomycin (10% FBS α-MEM) on two 10 cm culture dish. Non-adherent cells were isolated and cultured with M-CSF (30 ng/mL) in 10% FBS α-MEM on 10 cm Petri dishes for three days. Adhered cells were used as bone marrow derived macrophages (BMMs). We used the 96 well plate and cells were plated 1 × 10^4^ cells/well. BMMs were cultured with RANKL (10 ng/mL; R&D Systems, Minneapolis, MN, USA) and M-CSF (30 ng/mL) in 10% FBS α-MEM for 4 days in the presence of vehicle or BSC-W with 0.1% DMSO as solvent.

### 4.3. Tartrate-Resistant Acid Phosphatase (TRAP) Staining

The cells were fixed with formaldehyde (10%) for 5 min, permeabilized with Triton X-100 (0.1%) for 10 min, and then incubated with TRAP solution (Sigma-Aldrich, St. Louis, MO, USA) at room temperature for 10 min. Red stained cells with three or more nuclei were counted as mature osteoclasts.

### 4.4. Cytotoxicity Assay for Extracts of BSC-W

BMMs were incubated with M-CSF (30 ng/mL) in 10% FBS α-MEM in the presence of 0.1% DMSO or BSC-W for 3 days. Cell viability was assessed using a cell counting kit-8 (CCK-8; Dojindo Molecular Technologies, Kumamoto, JP) according to the manufacturer’s protocols.

### 4.5. Real-Time PCR

Real-time PCR was performed as previously described [26]. BMMs were cultured with RANKL (10 ng/mL) and M-CSF (30 ng/mL) in 10% FBS α-MEM for 0, 1, 2, or 3 days in the presence of 0.1% DMSO or BSC-W. For real-time PCR, primer sets were designed (Table 1) using the online primer3 program [45]. Total RNA was isolated using TRIzol reagent (Thermo Fisher Scientific Inc., Waltham, MA, USA) according to the manufacturer’s protocols. cDNA was synthesized using a M-MLV cDNA synthesis kit (Enzynomics, Daejeon, Korea) according to the manufacturer’s protocols. QPCR was conducted using the TOPreal qPCR 2× PreMIX (BioRad, Hercules, CA, USA) in a Real-Time PCR Detection System (BioRad, Hercules, CA, USA). The mRNA levels of the genes were analyzed by the 2^−ΔΔ*CT*^ method [46]. Glyceraldehyde-3-phosphate dehydrogenase (GAPDH) was used as an internal standard.

### 4.6. Western Blot

Western blotting was conducted as previously described [47]. Briefly, BMMs were incubated in the same manner as real-time PCR assays, after which cells were washed with phosphate-buffered saline (PBS) and lysed in lysis buffer (50 mM Tris-HCl, 150 mM NaCl, 5 mM EDTA, 1% Triton X-100, 1 mM sodium fluoride, 1 mM sodium vanadate, and 1% deoxycholate) containing protease inhibitors. Next, the supernatant containing proteins were subjected to 10% sodium dodecyl sulfate-polyacrylamide gel electrophoresis (SDS-PAGE). Separated proteins were subsequently transferred to a polyvinylidene difluoride (PVDF) membrane (Millipore Corporation, Billerica, MA, USA), after which membranes were blocked with 5% skim milk in TBST (10 mM Tris-HCl pH 7.5, 150 mM NaCl, and 0.1% Tween 20) for 1 h at room temperature. Blocked membranes were then incubated overnight at 4°C with a primary antibody, after which they were incubated with secondary antibody conjugated to horseradish peroxidase for 2 h at room temperature. Finally, the membranes were developed with CLARO^TM^ Mucho (bio-D, Gwangmyeong, Korea) using the LAS-4000 luminescent image analyzer (Fuji Photo Film Co. Ltd., Tokyo, JP).

### 4.7. Bone Pit Formation Assay

BMMs were cultured with RANKL (10 ng/mL) and M-CSF (30 ng/mL) in 10% FBS α-MEM for 3 days and then treated with vehicle (0.1% DMSO) or BSC-W for additional 3 days on an Osteo Assay Plate (24 well plate; Corning Inc., Corning, NY, USA) at a density of 6 × 10^4^ cells/well. Cells were removed with sodium hypochlorite (5%) for 5 min, after which the pit area was observed under a light microscope (magnification, 50×; Leica Microsystems, Wetzlar, Germany) and measured using the Image J software (NIH, Bethesda, MD, USA).

### 4.8. Statistical Analysis

All quantitative data are presented as the means ± standard deviations of three replicate experiments. The treatment groups were compared individually with the control group. Statistical differences were identified by Student’s t-tests and probability (*p*) values < 0.05 were considered significant (*p*-values * <0.05, ** <0.01, and *** <0.001).

## 5. Conclusions

The water extract of Boseokchal (BSC-W) inhibited osteoclast differentiation by blocking the expression of NFATc1 during RANKL-induced osteoclastogenesis. It was also confirmed that the expression levels of osteoclast differentiation/activation factors, such as TRAP, DC-STAMP, and cathepsin K, decreased with the decreased expression of NFATc1 by BSC-W. Therefore, BSC-W could be a useful functional food or a therapeutic agent for treatment of bone diseases such as osteoporosis.

## Figures and Tables

**Table 1:** Primer sequences used in this study.

Gene of Interest	Primer Sequence (5’→3’)	
	Sense	Anti-Sense
NFATc1	GGGTCAGTGTGACCGAAGAT	GGAAGTCAGAAGTGGGTGGA
c-Fos	CCAGTCAAGAGCATCAGCAA	AAGTAGTGCAGCCCGGAGTA
Cathepsin K	GGCCAACTCAAGAAGAAAAC	GTGCTTGCTTCCCTTCTGG
DC-STAMP	CCAAGGAGTCGTCCATGATT	GGCTGCTTTGATCGTTTCTC
TRAP	GATGACTTTGCCAGTCAGCA	ACATAGCCCACACCGTTCTC
GAPDH	AACTTTGGCATTGTGGAAGG	ACACATTGGGGGTAGGAACA
